# Hypothetical Estimands in Clinical Trials: A Unification of Causal Inference and Missing Data Methods

**DOI:** 10.1080/19466315.2022.2081599

**Published:** 2022-07-06

**Authors:** Camila Olarte Parra, Rhian M. Daniel, Jonathan W. Bartlett

**Affiliations:** a Department of Mathematical Sciences, University of Bath, Bath, UK; b Division of Population Medicine, Cardiff University, Cardiff, UK

**Keywords:** Causal inference, E9 addendum, Hypothetical estimand, Intercurrent events, Missing data

## Abstract

The ICH E9 addendum introduces the term intercurrent event to refer to events that happen after treatment initiation and that can either preclude observation of the outcome of interest or affect its interpretation. It proposes five strategies for handling intercurrent events to form an estimand but does not suggest statistical methods for estimation. In this article we focus on the hypothetical strategy, where the treatment effect is defined under the hypothetical scenario in which the intercurrent event is prevented. For its estimation, we consider causal inference and missing data methods. We establish that certain “causal inference estimators” are identical to certain “missing data estimators.” These links may help those familiar with one set of methods but not the other. Moreover, using potential outcome notation allows us to state more clearly the assumptions on which missing data methods rely to estimate hypothetical estimands. This helps to indicate whether estimating a hypothetical estimand is reasonable, and what data should be used in the analysis. We show that hypothetical estimands can be estimated by exploiting data after intercurrent event occurrence, which is typically not used. Supplementary materials for this article are available online.

## 1 Introduction

The analysis of randomized trials is often complicated by the occurrence of certain events that affect the interpretation of the treatment effect or preclude the observation of the outcome of interest. Such occurrences, termed “intercurrent events” (ICE) by the recently-published ICH E9 addendum on estimands (International Council for Harmonisation of Technical Requirements for Pharmaceuticals for Human Use 2019), include treatment discontinuation, addition of rescue medication, or death prior to measurement of the outcome of interest. In the presence of such ICEs, the importance of clear specification of a trial’s treatment effect “estimand” and how the statistical analysis targets this estimand has been increasingly recognized over the last decade.

The U.S. National Research Council report on the Prevention and Handling of Missing Data in Clinical Trials highlighted the importance of trials clearly specifying the target estimand(s), and how the trial design and statistical analysis should be chosen to support its reliable estimation (National Research Council 2010). Since then, a number of authors have considered the complex questions involved in how to choose and specify an estimand and how to select an appropriate statistical method to estimate it (Mallinckrodt et al. 2012, 2019, 2020; Carpenter, Roger, and Kenward 2013; Holzhauer, Akacha, and Bermann 2015).

According to the ICH E9 framework, strategies for dealing with intercurrent events must be specified when choosing and defining the target estimand of a clinical trial. The addendum does not however specify how these might or should correspond to statistical analysis methods. One of the proposed strategies is labeled as hypothetical. Under the hypothetical strategy, the causal effect is targeting what would have happened if the ICE had (somehow) been prevented from occurring. For instance, if rescue medication was not available or patients could be prevented from discontinuing treatment. For patients in the trial for whom the ICE did not occur, their observed outcome corresponds to the outcome of interest under the hypothetical strategy, whereas for those who experienced the ICE, the potential outcome of interest is missing. Consequently, the existing literature has almost exclusively focused on tackling the problem of estimation of hypothetical estimands from the perspective of missing data, by deleting any outcomes observed after ICE occurrence and applying methods such as direct likelihood (e.g., using linear mixed models) or multiple imputation (Qu et al. 2020).

Until recently (Lipkovich, Ratitch, and Mallinckrodt 2020; Bowden et al. 2020; Qu et al. 2020; Michiels et al. 2021; Qu, Luo, and Ruberg 2021), relatively little has been published on the topic of estimation of estimands from the perspective of modern casual inference. Indeed, perhaps surprisingly, the ICH E9 addendum itself does not explicitly mention causal inference concepts or methods, although these are clearly relevant.

The hypothetical strategy has been used when the ICE is addition of rescue medication. A recent systematic review on rescue medication in published trials of asthma and eczema found that its use was not routinely reported or accounted for, even when there was evidence of an imbalance in rescue medication between arms (Ster, Cornelius, and Cro 2020). When analyses aiming to account for rescue medication were reported, the authors of the review considered that they were mainly targeting a hypothetical estimand with suboptimal methods and concluded that further guidance was warranted.

In diabetes trials, rescue medication is usually available for ethical reasons. An example of such a trial compared dapagliflozin, dapagliflozin plus saxagliptin, and glimepiride in patients with type 2 diabetes who were using metformin (Müller-Wieland et al. 2018). Insulin therapy was available as rescue medication. The analysis of the primary endpoint was performed using a linear mixed model with fixed effects for treatment group and covariates after deleting values beyond the first use of rescue medication. Here, we will discuss alternative approaches and their corresponding underlying assumptions, using either only the values prior to rescue medication or the full observed data including values after the ICE occurrence and how they relate to this approach.

In this article, we review concepts from causal inference to characterize precisely the conditions under which hypothetical estimands can be estimated from trial data. We describe statistical estimators of these arising from both the causal inference and missing data literatures, and establish that for each missing data estimator there is a corresponding numerically identical causal inference estimator, thereby unifying the sets of methods.

We begin in Section 2 with a review of the concepts and tools in causal inference, first for a setting with a treatment decision at a single time point, and then for a generalized setting where treatment changes can occur at multiple times. In Section 3, we consider the definition and estimation of a hypothetical estimand in a simplified setting in which the ICE can only occur at a single time point, linking it to the concepts and methods reviewed in Section 2. In Section 4, we consider the more general setup in which an ICE can occur at multiple time points. Finally, we give conclusions in Section 5.

## 2 A Brief Review of Causal Inference Concepts, Assumptions, and Estimators

In this section, we review the key concepts, assumptions and estimation methods from causal inference for studies with time-varying treatments, drawing on Hernan and Robins (2020), Robins and Hernán (2009), Tsiatis et al. (2020), Daniel et al. (2013), and Ding and Li (2018).

### 2.1 Identification Assumptions

#### 2.1.1 Time-Fixed Treatment

Clinical trials usually compare two or more treatments for a given condition and evaluate their effects on an outcome of interest. The potential outcomes framework provides a formal definition for such causal effects and the assumptions required to estimate them (Rubin 1974). “Potential outcome” refers to the response that would have been observed on a patient had they been assigned a particular treatment. Thus, there is a potential outcome for each patient for every treatment we might feasibly assign to them. Except in certain special situations, patients only receive one treatment and therefore only one of their potential outcomes is observed.

The potential outcome, denoted *Y^a^*, expresses the outcome *Y* under assignment to treatment *a*. We can then define the target causal effect of interest (the estimand) as a contrast of the distributions of such potential outcomes. For a dichotomous treatment, *A*, we may for example be interested in the mean difference, $E(Y^{a=1})-E(Y^{a=0})$ , or simply $E(Y^{1})-E(Y^{0})$ .

In RCTs, the treatment at baseline is assigned at random but the occurrence of the ICE is not. As we will see in the next section, the ICE can be considered a treatment that is not randomly assigned. The causal effect of interest can be estimated if certain *identifiability assumptions* are satisfied. First, the interventions have to be sufficiently well defined to ensure *consistency*, which states that the observed outcome corresponds to the potential outcome under the treatment received: $Y=Y^{a}$ if *A* = *a*, where *A* denotes the variable recording the treatment a given patient receives (VanderWeele 2009). Consider an oncology trial where we compare chemotherapy versus no chemotherapy. The chemotherapy treatment would be considered ill-defined if the type of chemotherapy and regimen are not specified. Also no chemotherapy can imply no treatment or follow up at all or just standard of care or many other options. As we would not expect that different types of chemotherapy regimens would yield similar outcomes, then the potential outcomes *Y^a^* are not sufficiently well defined.

As patients can usually receive only one treatment, we compare different groups of patients receiving the different treatments of interest. Randomization ensures that the different groups of patients have similar prognostic factor distributions. In the absence of randomization, the effect estimate has to be adjusted for a sufficient set of confounders to ensure that patients are comparable in terms of their prognostic factors. This is the second identifiability condition known as *conditional exchangeability*. In other words, each of the potential outcomes *Y^a^* for the different possible values of *a* has to be independent of the actual treatment received *A*, given the confounders *L*: $Y^{a}⊥⊥A|L$ .

Finally, the last identifiability assumption is *positivity*. This means that for every patient, on the basis of their confounder values *L*, there is a nonzero probability that they could receive each of the treatments under study (Petersen et al. 2012). It would not be sensible to consider patients who, on the basis of one or more of their confounder values, would always receive a given treatment. This could happen if, say, a particular confounder level implies contraindication for one of the treatments. Therefore, all patients should have a nonzero probability of receiving the different treatments P(A=a|L=l)>0 for all values of *a* and *l* such that P(L=l)>0 .

**Fig. 1:**
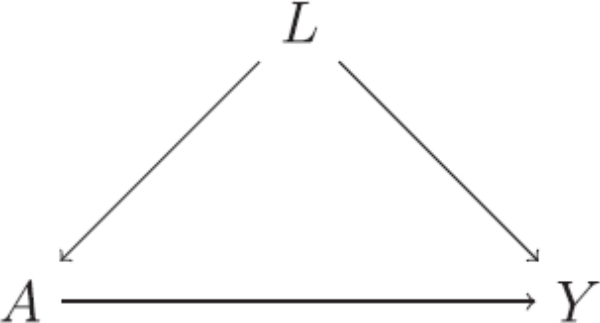
Example of a direct acyclic graphs (DAG) relating treatment *A*, outcome *Y* and confounders *L.*

Directed acyclic graphs (DAGs) are a useful tool for encoding causal assumptions. These graphs are composed of nodes that represent random variables, including treatment, outcome and covariates, and edges connecting the nodes (Figure 1). DAGs are said to be directed because the edges have arrows indicating the direction of the causal effect and acyclic because a directed path that is, edge or series of consecutive edges, cannot lead back to an initial node. The absence of an edge between two nodes encodes the assumption that there is no direct causal effect between them. In Figure 1, we would omit the edge L→A in an RCT to show that *A* is assigned at random. Briefly, paths between nodes are made up of (1) chains (e.g., L→A→Y ), (2) forks (e.g., A←L→Y ) and (3) inverted forks (e.g., A→Y←L ). The variable at the center of an inverted fork, in this case *Y*, is known as a *collider* on that path. Associations are transmitted along chains and forks but not along inverted forks; thus, paths consisting only of chains and forks are called open paths, but those containing at least one inverted fork are closed. The association transmitted along an open path can be blocked by conditioning on a central variable in a chain or fork, but a closed path is unblocked by conditioning on a collider, and correspondingly a conditional association induced. The composition of these 3-variable phenomena lead to an algorithm for deciding whether or not a set of covariates is sufficient to control for confounding. For example, in Figure 1, were the arrow from *A* to *Y* to be removed, *A* and *Y* would be marginally associated (via *L*) but conditionally independent given *L*, which indicates that adjusting for *L* is sufficient to control for confounding under the assumptions of this simple DAG.

#### 2.1.2 Time-Varying Treatment

**Fig. 2:**
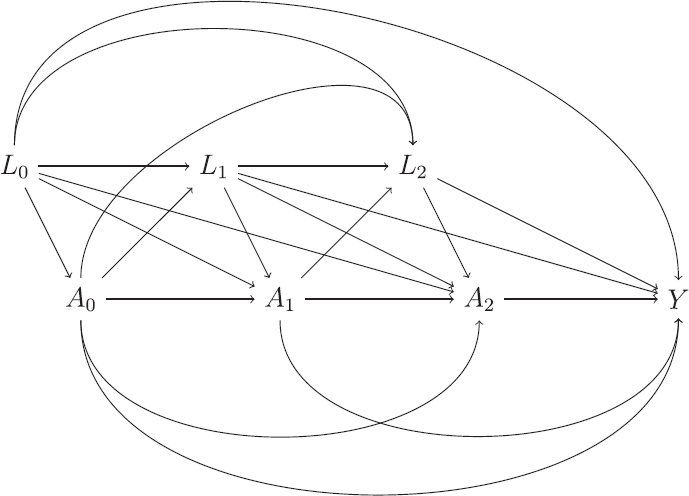
Directed acyclic graph (DAG) of a study with time-varying treatment.

The preceding setup can be extended to a more general one where treatment can change over time. Figure 2 shows a possible DAG for a study where treatment can change at three time points, with $A_{0},A_{1},A_{2}$ denoting variables for treatment at each time. The treatment at a given time point *k* can depend on the earlier treatment values and also the earlier values of the variables *L*. The final outcome of interest is denoted *Y*.

In the setting with time-varying treatments we are typically interested in comparing different treatment regimes, that is different (hypothetical) ways of assigning treatments. A static treatment regime is one in which the decision on which treatment to assign at each time point does *not* depend on the time-varying variables *L*, whereas dynamic regimes are those where the treatment decisions can be based on the hypothetical values of *L* that would be observed under that regime. As we will see later, the hypothetical estimand of “no ICE” corresponds to a static regime. For the DAG in Figure 2 a static regime is defined by specifying particular values for the three treatment variables. For example, assuming there are two treatments available, coded 0 and 1, a particular regime is $\bar{a}=(0,0,0)$ , which corresponds to assigning the first treatment at each of the three time points. The potential outcome under this regime is denoted $Y^{\bar{a}}$ .

The identifiability conditions described previously for the setting with a single treatment assignment can be extended to this more general setting with time-varying treatments. The no unmeasured confounding or exchangeability condition has a number of different versions in the time-varying treatment setting. For our purposes we use the following version, which states

(1) $$Y^{\bar{a}}⊥⊥A_{k}|{\bar{A}}_{k-1}={\bar{a}}_{k-1},{\bar{L}}_{k}$$

for k=0,1,…,K and all static treatment regimes $\bar{a}$ , where ${\bar{A}}_{k-1}=(A_{0},\ldots ,A_{k-1})$ denotes the history of treatment received through to time *k* – 1, and similarly for ${\bar{a}}_{k-1}$ and ${\bar{L}}_{k}$ . This condition is satisfied if treatment assignment at each time point depends only on previous treatments and measured time-varying confounders, as in the DAG in Figure 2. We will see later that this assumption plays a critical role regarding which variables are included in *L_k_* in order to provide valid inferences.

The positivity condition is similarly extended in the time-varying treatment setting to the following

(2) $$\begin{array}{c} P(A_{k}=a_{k}|{\bar{A}}_{k-1}={\bar{a}}_{k-1},{\bar{L}}_{k}={\bar{l}}_{k})>0\;\text{for}\;\text{all}\;\\ \;\;(a_{k},{\bar{a}}_{k-1},{\bar{l}}_{k})\;\text{that}\;\text{satisfy}\;f_{{\bar{A}}_{k-1},{\bar{L}}_{k}}({\bar{a}}_{k-1},{\bar{l}}_{k})>0 \end{array}$$

for each *k*. In words, this says that for all combinations of treatment histories and time-varying confounders up to time *k* – 1 that can occur in the study, there is a positive probability of each of the possible treatments being given at time *k*. In fact, if we are interested in a particular treatment strategy, this condition only needs to hold for treatment histories compatible with the strategy of interest (see Technical Point 19.2 of Hernan and Robins 2020). For example, if we are interested in the treatment strategy of giving treatment zero throughout, $\bar{a}=(0,\ldots ,0)$ , we require only that

$$\begin{array}{c} P(A_{k}=0|{\bar{A}}_{k-1}={\bar{0}}_{k-1},{\bar{L}}_{k}={\bar{l}}_{k})>0\;\text{for}\;\text{all}\;{\bar{l}}_{k}\\ \;\text{that}\;\text{satisfy}\;f_{{\bar{A}}_{k-1},{\bar{L}}_{k}}({\bar{0}}_{k-1},{\bar{l}}_{k})>0 \end{array}$$

for each *k*. We will see later that the positivity assumption plays an important role in the feasibility of estimation of hypothetical estimands where whether an ICE occurs depends deterministically on time-varying confounders.

In what follows, where there is no ambiguity introduced, we use f(x|a,b,c) as shorthand for $f_{X|A,B,C}(x|a,b,c)$ . In the case that *X* is discrete, f(x|a,b,c)=P(X=x|A=a,B=b,C=c) .

### 2.2 Estimation Methods

#### 2.2.1 G-Formula

We now review the two most commonly adopted approaches for estimation of the causal effects of treatment in the time-varying treatment setting. The first is G-formula or G-computation. Under the previously stated identification conditions, for a given treatment regime $\bar{a}$ the density function of the potential outcomes under this regime can be shown (sec. 5.4 of Tsiatis et al. 2020) to be equal to

(3) $$\begin{array}{c} f_{Y^{\bar{a}}}(y)=\int_{l_{0}}\int_{l_{1}}\int_{l_{2}}f_{Y|\bar{A},\bar{L}}(y|\bar{a},\bar{l})f_{L_{2}|{\bar{A}}_{1},{\bar{L}}_{1}}(l_{2}|{\bar{a}}_{1},{\bar{l}}_{1})\\ \;\;f_{L_{1}|A_{0},L_{0}}(l_{1}|a_{0},l_{0})f_{L_{0}}(l_{0})dl_{2}dl_{1}dl_{0}, \end{array}$$

where we have taken *K* = 2 for concreteness, $\bar{a}=(a_{0},a_{1},a_{2})$ and ${\bar{a}}_{1}=(a_{0},a_{1})$ . Often we will be interested in the mean outcome under a given treatment regime, which can then be shown to equal

(4) $$\begin{array}{c} E(Y^{\bar{a}})=\int_{l_{0}}\int_{l_{1}}\int_{l_{2}}E(Y|\bar{a},\bar{l})f(l_{2}|{\bar{a}}_{1},{\bar{l}}_{1})f(l_{1}|a_{0},l_{0})f(l_{0})dl_{2}dl_{1}dl_{0}\\ =E\left(E\left[E\left\{E(Y|\bar{A}=\bar{a},\bar{L})|{\bar{A}}_{1}={\bar{a}}_{1},{\bar{L}}_{1}\right\}|A_{0}=a_{0},L_{0}\right]\right). \end{array}$$

To implement G-formula we can specify and fit models for the conditional distributions

(5) $$\begin{array}{c} f(y|\bar{a},\bar{l}),\\ f(l_{k}|{\bar{a}}_{k-1},{\bar{l}}_{k-1}),\;k=1,\ldots ,K,\\ f(l_{0}). \end{array}$$

If we are only interested in the mean outcome (as opposed to other aspects of the distribution) under the treatment strategy, then the conditional model for *Y* can be replaced with a model for its conditional expectation. When the *L_k_* are multivariate, these regressions become multivariate, which is more difficult, particularly if the components of *L_k_* are a mixture of continuous and discrete variables. An issue for this approach is that it is not usually clear which order should be chosen. We note that a similar issue arises in the specification of imputation models for multivariate missing data (Erler, Rizopoulos, and Lesaffre 2019).

Having specified and fitted the models, the G-formula identification Equations (3) or (4) can be used. However, evaluation of the integrals involved in general is difficult. To circumvent this, a Monte Carlo integration approach can be used, in which the values of the time-varying confounders *L_k_* are simulated from the fitted models sequentially. For further details, see, Daniel, De Stavola, and Cousens (2011).

The resulting G-formula estimates of $E(Y^{\bar{a}})$ are consistent provided the previously stated identification assumptions hold and the models for the conditional distributions of the time-varying confounders and the model for the outcome (5), are correctly specified.

#### 2.2.2 Inverse Probability of Treatment Weighting

A different approach is to use inverse probability of treatment weighting to create a pseudopopulation in which the time-varying treatment assignment is independent of the values of the (time-varying) covariates. In other words, we create a pseudopopulation in which there are no longer arrows into the treatment nodes from any other node, but all other relationships remain unaltered. This is achieved by weighting each patient by the inverse of the probability of receiving the treatment they in fact received at each time point given the covariate and treatment history. This is the time-varying treatment extension of the propensity score (the probability of treatment given covariates). In the time-varying treatment setting, the (unstabilized) weight for patient *i* is

(6) $$W_{i}=\prod_{k=0}^{K}\frac{1}{f(A_{k,i}|{\bar{A}}_{k-1,i},{\bar{L}}_{k,i})},$$

where patient *i*’s treatment history is $\left(A_{0,i},A_{1,i},\ldots ,A_{K,i}\right)$ , their time-varying confounder history is $\left(L_{0,i},L_{1,i},\ldots ,L_{K,i}\right)$ , and ${\bar{A}}_{-1,i}$ is taken to be zero for all *i* by definition. The potential mean outcome under the treatment strategy of interest can then be estimated as the weighted average of the outcomes among those patients whose treatment history matches the treatment strategy $\bar{a}$ we are targeting:

(7) $$\frac{\sum_{i=1}^{n}I({\bar{A}}_{i}=\bar{a})W_{i}Y_{i}}{\sum_{i=1}^{n}I({\bar{A}}_{i}=\bar{a})W_{i}}$$

where I(·) denotes the indicator function.

In practice, the conditional distributions of the time-varying treatment variables involved in the definition of the weights are not known, but must instead be estimated. When there are only two treatment options at each time, such that each *A_k_* is binary, these can be logistic regression models. The resulting estimator of $E(Y^{\bar{a}})$ is consistent provided these models are correctly specified, along with the same identification assumptions stated previously.

## 3 Definition and Estimation of a Hypothetical Estimand in a Simplified Setting

In this section, we apply the assumptions and methods introduced in Section 2 to the problem of estimating a hypothetical estimand in a randomized trial in which the ICE can take place at only a single fixed time point. We then compare and contrast the causal inference estimators with missing data estimators which are currently more commonly adopted in practice for the estimation of hypothetical estimands.

**Fig. 3:**
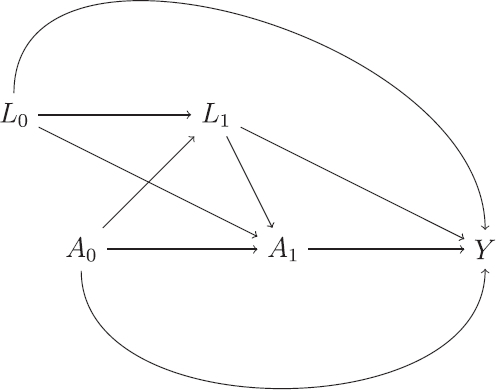
Directed acyclic graph (DAG) representation of a randomized trial where the ICE occurring can only occur at a fixed time point. *A*_0_ denotes randomized treatment, *A*_1_ occurrence of ICE, *L*_0_ baseline variables, *L*_1_ post-baseline variables measured before the occurence of the ICE and *Y* final outcome.

We consider inference under the setup depicted by the DAG in Figure 3, which is a special case of the general time-varying treatment setting described in Section 2.1.2. Here *A*_0_ represents randomized treatment at baseline. Unlike Figure 2, there is no arrow from *L*_0_ to *A*_0_ due to the fact that treatment at baseline is randomly assigned. The second “treatment” variable *A*_1_ represents whether or not the ICE occurs for a particular patient. The ICE can be considered a “treatment” because we want to intervene on it to set it to 0.

The potential outcomes $Y^{a_{0},a_{1}}$ denote the outcome were we to assign treatment *a*_0_ and intervene (somehow) on the ICE to set it to level *a*_1_. The effect of treatment can be expressed as the expected outcome of assigning the full population to treatment 1 versus assigning them to treatment 0 in the hypothetical scenario were the ICE prevented from occurring:

(8) $$E(Y^{1,0})-E(Y^{0,0}).$$

In some cases, the post-baseline variables (*L*_1_) can include measurements of the outcome at intermediate visits. For instance in a diabetes trial, *L*_1_ might consist of measurements of glycemic control, like glycated hemoglobin (HbA1c) and fasting plasma glucose (FPG), assessed at an intermediate follow-up visit, while *Y* denotes HbA1c at the second (final) visit.

### 3.1 Causal Inference Estimation Approaches

The sequential exchangeability assumption (Equation (1)) holds here, given covariates *L*_0_, *L*_1_, since the DAG in Figure 3 is a special case of the general DAG in Figure 2. Informally this can be seen to hold here because the treatment *A*_0_ is randomly assigned and the ICE *A*_1_ (which is analogous to the “second treatment”) is only influenced by assigned treatment *A*_0_ and the measured variables *L*_0_ and *L*_1_. Thus, $Y^{a_{0},a_{1}=0}⊥⊥A_{0}$ and $Y^{a_{0},a_{1}=0}⊥⊥A_{1}|A_{0},L_{0},L_{1}$ . We emphasize that *L*_0_ and *L*_1_ consist of all variables which influence the ICE occurrence (*A*_1_) and the outcome *Y*. In particular, it is not sufficient to specify *L*_0_ and *L*_1_ as simply the baseline and intermediate measures of the outcome variable unless these are truly the only variables influencing the occurrence of the ICE and outcome.

For the positivity assumption (Equation (2)), first since in randomized trials $P(A_{0}=0)=P(A_{0}=1)=0.5$ , it is clearly the case that $P(A_{0}=a_{0}|L_{0}=l_{0})=P(A_{0}=a_{0})>0$ for both $a_{0}=0$ and $a_{0}=1$ . Second, the occurrence of the ICE should also satisfy that $P(A_{1}=0|A_{0}=a_{0},L_{0}=l_{0},L_{1}=l_{1})>0$ for all possible *l*_0_ and *l*_1_ values in both treatment arms. This means that for all possible combinations of *L*_0_, *L*_1_ and *A*_0_, the probability of *not* having the ICE must be nonzero. Note that as we are only interested in the potential outcomes in the absence of the ICE, then $P(A_{1}=1|A_{0}=a_{0},L_{0}=l_{0},L_{1}=l_{1})>0$ is not required, which implies that for some values of *a*_0_, *l*_0_ and *l*_1_ it can be that $P(A_{1}=0|A_{0}=a_{0},L_{0}=l_{0},L_{1}=l_{1})=1$ without representing a violation of the positivity assumption.

#### 3.1.1 G-formula

From Equation (4) the G-formula estimator for the mean potential outcome under a general treatment regime $\bar{a}$ is given by

$$E(Y^{\bar{a}})=\int_{l_{0}}\int_{l_{1}}E(Y|\bar{a},\bar{l})f(l_{1}|a_{0},l_{0})f(l_{0})\;dl_{1}\;dl_{0}.$$

Unlike in the general observational study setting, in our setting the first treatment *A*_0_ is randomly assigned. This means in particular that $A_{0}⊥⊥L_{0}$ , which in turn means that $f(l_{0})=f(l_{0}|a_{0})$ . Thus, the preceding equation can in our setting be written as

$$\begin{array}{c} E(Y^{\bar{a}})=\int_{l_{0}}\int_{l_{1}}E(Y|\bar{a},\bar{l})f(l_{1}|a_{0},l_{0})f(l_{0}|a_{0})\;dl_{1}\;dl_{0}\\ =\int_{l_{0}}\int_{l_{1}}E(Y|\bar{a},\bar{l})f(l_{1},l_{0}|a_{0})\;dl_{1}\;dl_{0}. \end{array}$$

To use this to estimate $E(Y^{\bar{a}})$ we could specify models for $E(Y|\bar{a},\bar{l})$ and $f(l_{1},l_{0}|a_{0})$ . To avoid having to specify a model, we can use the empirical distribution of *L*_0_ and *L*_1_ among those randomized to $A_{0}=a_{0}$ . This motivates the G-formula estimator:

(9) $$\hat{E}(Y^{\bar{a}})=\frac{\sum_{i=1}^{n}I(A_{0,i}=a_{0})\hat{E}(Y_{i}|a_{0},a_{1},L_{0,i},L_{1,i})}{\sum_{i=1}^{n}I(A_{0,i}=a_{0})}$$

which relies only on a model for $E(Y|\bar{a},\bar{l})$ , that is, an appropriate model for the mean of *Y* with *A*_0_, *A*_1_, *L*_0_, and *L*_1_ as covariates, noting that our estimator then requires predictions from this model where *A*_1_ is set to *a*_1_. If *Y* is continuous we might for example choose a linear regression model with main effects of randomized treatment *A*_0_, occurrence of ICE *A*_1_, *L*_0_, and *L*_1_. This model would make use of all the observed data, including outcomes *Y* which take place after an ICE. Through the inclusion of *A*_1_ as a covariate, it models how the ICE influences the final outcome *Y*. When the ICE is receipt of rescue medication, this corresponds to use of the post-rescue outcomes with adjustment for rescue, and this type of approach has been discussed previously by Holzhauer, Akacha, and Bermann (2015).

As described previously, the hypothetical estimand corresponds to the contrast of the treatment regimes $\bar{a}=(1,0)$ and $\bar{a}=(0,0)$ , where we set *A*_1_ to 0. A G-formula estimator for the hypothetical estimand under no ICE is thus,

(10) $$\hat{E}(Y^{a_{0},a_{1}=0})=\frac{\sum_{i=1}^{n}I(A_{0,i}=a_{0})\hat{E}(Y_{i}|a_{0},a_{1}=0,L_{0,i},L_{1,i})}{\sum_{i=1}^{n}I(A_{0,i}=a_{0})}.$$

The G-formula estimator is thus predicting, for every patient randomized to treatment group *a*_0_, what their outcome would be were the ICE set to not occur.

Since here we are only interested in regimes which set $a_{1}=0$ , we do not need in fact to model how occurrence of the ICE influences *Y*, since we only need to predict outcomes under no ICE $a_{1}=0$ . Thus, an alternative G-formula approach for the hypothetical estimand is to only specify and fit a model among those patients who did not experience an ICE ($A_{1}=0$ ), that is, for $E(Y|A_{0}=a_{0},A_{1}=0,L_{0},L_{1})$ . For example if we are interested in the potential outcome under a particular value of the treatment *A*_0_ = *a*_0_ we might assume that $E(Y|A_{0}=a_{0},A_{1}=0,L_{0},L_{1})=\beta_{0}^{a_{0}}+\beta_{1}^{a_{0}}L_{0}+\beta_{2}^{a_{0}}L_{1}$ . This model can be fitted by ordinary least squares to those randomized to $A_{0}=a_{0}$ and for whom $A_{1}=0$ , giving estimates ${\hat{\beta }}_{0}^{a_{0}}$ , ${\hat{\beta }}_{1}^{a_{0}}$ , and ${\hat{\beta }}_{2}^{a_{0}}$ . The G-formula estimator is then equal to

(11) $$\hat{E}(Y^{a_{0},a_{1}=0})=\frac{\sum_{i=1}^{n}I(A_{0,i}=a_{0})\left\{{\hat{\beta }}_{0}^{a_{0}}+{\hat{\beta }}_{1}^{a_{0}}L_{0,i}+{\hat{\beta }}_{2}^{a_{0}}L_{1,i}\right\}}{\sum_{i=1}^{n}I(A_{0,i}=a_{0})}.$$

The approach which specifies and fits a model to only those patients who did not experience the ICE makes fewer assumptions than the one which specifies and fits a model to all observed data. The approach which uses all the data has the potential to give improved precision of estimates (and hence greater power), but is more vulnerable to model misspecification. Similarly there is a tradeoff between precision and model misspecification when fitting a single model and including the randomized treatment *A*_0_ as covariate or fitting separate models by treatment arm to relax modeling assumptions. The different modeling alternatives can also be combined for example, separate models by treatment arm among ICE-free.

#### 3.1.2 Inverse Probability of Treatment Weighting

We now apply the general IPW estimator described in Section 2.2.2. Applied to the current setting, the IPW estimator of the mean of $Y^{a_{0},a_{1}=0}$ from Equation (7) is given by

(12) $$\frac{\sum_{i=1}^{n}I({\bar{A}}_{i}=(a_{0},0))W_{i}Y_{i}}{\sum_{i=1}^{n}I({\bar{A}}_{i}=(a_{0},0))W_{i}}.$$

This is a weighted mean of the outcomes from those patients who were randomized to treatment *a*_0_ and in whom the ICE did not occur. Under 1:1 randomization $P(A_{0}=a_{0}|L_{0})=0.5$ , and so the weights *W_i_* are given by

(13) $$\begin{array}{c} W_{i}=\frac{1}{0.5}\times \frac{1}{f(A_{1,i}|A_{0,i},L_{0,i},L_{1,i})}\\ =\frac{2}{f(A_{1,i}|A_{0,i},L_{0,i},L_{1,i})}. \end{array}$$

Since the occurrence of the ICE is typically not under the investigator’s full control, we must postulate and fit a model for $P(A_{1}=0|A_{0},L_{0},L_{1})$ . For example, we could fit a logistic regression for *A*_1_ with *A*_0_, *L*_0_, and *L*_1_ as covariates. Here the logistic models to estimate the weights could also be fitted separately by treatment arm. As with G-formula, there is a similar trade off of making the models less vulnerable to model misspecification but at the expense of precision.

### 3.2 Missing Data Approaches

#### 3.2.1 Likelihood Based Missing Data Approaches

As described in Section 1, currently a commonly adopted estimation approach when targeting hypothetical estimands with continuous endpoints is to fit a linear mixed model to the repeated measures of the outcome variable using maximum likelihood, after deleting any outcome measurements which were made after the ICE took place. These are based on assuming the resulting missing data are missing at random (MAR).

**Fig. 4:**
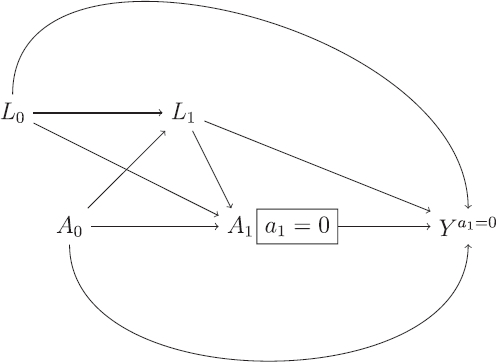
Single-world intervention graph (SWIG) resulting from the DAG shown in Figure 3, intervening to set the ICE to $a_{1}=0$ .

We will use the theory of DAGs to check whether MAR holds for the missing hypothetical outcomes under the DAG shown in Figure 3. To do so, we make use of an extension of DAGs: single world intervention graphs (SWIGs), as described by Richardson and Robins (2013). A SWIG takes as its input a DAG, and shows the graph that would result under an intervention which fixes the values of certain variables. Figure 4 shows the SWIG that results if we intervene to set the ICE $a_{1}=0$ . Each variable intervened on is split into two parts, the first (in capitals) which denotes the original variable, taking whatever value it would naturally take (without intervention), and the second part (lower case) indicating the intervened value. Variables which are affected by those variables intervened on are changed to their potential outcome value under the specified values of the intervened variables. Thus, when we intervene to set $a_{1}=0$ , *Y* becomes $Y^{a_{1}=0}$ .

Unlike the DAG, the SWIG in Figure 4 contains the partially observed potential outcomes $Y^{a_{1}=0}$ of interest under the hypothetical estimand. To check MAR, we note that the indicator of missingness in the hypothetical outcomes $Y^{a_{1}=0}$ is identical to the ICE variable *A*_1_, since it is those individuals with $A_{1}=1$ for which $Y^{a_{1}=0}$ is missing. MAR here means that the missingness indicator *A*_1_ is independent of the partially observed $Y^{a_{1}=0}$ conditional on *A*_0_, *L*_0_ and *L*_1_. This conditional independence can be read off from the SWIG, since once we condition on *A*_0_, *L*_0_ and *L*_1_ there are no open paths from *A*_1_ to $Y^{a_{1}=0}$ . Analogous to the sequential exchangeability assumption, we emphasize that we must ensure the variables used as *L*_0_ and *L*_1_ in estimation do include all common causes of the ICE *A*_1_ and the outcome *Y*. For example, while *L*_0_ and *L*_1_ may typically need to include measurements of the outcome variable at baseline and the intermediate time point, there will generally be additional common causes of the ICE and outcome *Y*, and these must be included in *L*_0_ and *L*_1_ for the DAG in Figure 3 to be correct.

Since the potential outcomes $Y^{a_{1}=0}$ which are of interest for the hypothetical estimand are MAR given *A*_0_, *L*_0_ and *L*_1_, it follows that an observed data likelihood analysis assuming the missingness is ignorable will give consistent estimates in this scenario under the previously stated assumptions and provided the full data model assumed is correctly specified. These conclusions are in agreement with those of Holzhauer, Akacha, and Bermann (2015), who proposed fitting a joint mixed effects model for $L_{0},L_{1},Y$ conditional on *A*_0_, after deleting post ICE outcomes, or alternatively use of multiple imputation to impute $Y^{a_{1}=0}$ in those with $A_{1}=1$ , again ensuring that $L_{0},L_{1},A_{0}$ are included in the imputation model.

We now show that particular observed data likelihood based estimators are identical to particular G-formula estimators. Consider the data on *L*_0_, *L*_1_ and *Y* in treatment group $A_{0}=a_{0}$ , after deleting any *Y* values for individuals with $A_{1}=1$ . Suppose that *L*_1_ is a single variable and that we fit a bivariate normal model (a type of linear mixed model) to the resulting $\left(L_{1},Y^{a_{1}=0}\right)$ data in group $A_{0}=a_{0}$ assuming MAR, with the means of *L*_1_ and $Y^{a_{1}=0}$ depending linearly on *L*_0_ but with separate coefficients, and an unstructured covariance matrix. The bivariate normal model implies that

$$\begin{array}{c} E(Y^{a_{1}=0}|A_{0}=a_{0},L_{0},L_{1})=\beta_{20}^{a_{0}}+\beta_{21}^{a_{0}}L_{0}+\beta_{22}^{a_{0}}L_{1}\\ E(L_{1}|A_{0}=a_{0},L_{0})=\beta_{10}^{a_{0}}+\beta_{11}^{a_{0}}L_{0}. \end{array}$$

The observed data likelihood function under MAR factorizes (sec. 7.2 of Little and Rubin 2019) such that the MLEs of the parameters in these two models are obtained by fitting the $Y^{a_{1}=0}$ model among those with $Y^{a_{1}=0}$ observed (here meaning $A_{1}=0$ ) and for the *L*_1_ model using all patients. Then we have that

$$\begin{array}{c} E(Y^{a_{1}=0}|A_{0}=a_{0},L_{0})=E\left\{E(Y^{a_{1}=0}|A_{0}=a_{0},L_{0},L_{1})|A_{0}=a_{0},L_{0}\right\}\\ =E(\beta_{20}^{a_{0}}+\beta_{21}^{a_{0}}L_{0}+\beta_{22}^{a_{0}}L_{1}|A_{0}=a_{0},L_{0})\\ =\beta_{20}^{a_{0}}+\beta_{21}^{a_{0}}L_{0}+\beta_{22}^{a_{0}}(\beta_{10}^{a_{0}}+\beta_{11}^{a_{0}}L_{0}). \end{array}$$

Taking expectations of this conditional on *A*_0_ = *a*_0_ we have

$$\begin{array}{c} E(Y^{a_{1}=0}|A_{0}=a_{0})=\beta_{20}^{a_{0}}+\beta_{21}^{a_{0}}E(L_{0}|A_{0}=a_{0})\\ \;+\beta_{22}^{a_{0}}(\beta_{10}^{a_{0}}+\beta_{11}^{a_{0}}E(L_{0}|A_{0}=a_{0})). \end{array}$$

Taking $\hat{E}(L_{0}|A_{0}=a_{0})=\frac{\sum_{i=1}^{n}I(A_{0,i}=a_{0})L_{0,i}}{\sum_{i=1}^{n}I(A_{0,i}=a_{0})}$ as the nonparametric MLE of $E(L_{0}|A_{0}=a_{0})$ , by the invariance property of MLE the MLE of $E(Y^{a_{1}=0}|A_{0}=a_{0})$ is given by

$$\begin{array}{c} \hat{E}(Y^{a_{1}=0}|A_{0}=a_{0})={\hat{\beta }}_{20}^{a_{0}}+{\hat{\beta }}_{21}^{a_{0}}\hat{E}(L_{0}|A_{0}=a_{0})\\ \;+{\hat{\beta }}_{22}^{a_{0}}({\hat{\beta }}_{10}^{a_{0}}+{\hat{\beta }}_{11}^{a_{0}}\hat{E}(L_{0}|A_{0}=a_{0})). \end{array}$$

The model for *L*_1_ is fitted to all those with *A*_0_ = *a*_0_. A property of ordinary least squares estimators is that the sample mean of the dependent variable (here *L*_1_) is equal to the predicted value of the dependent variable when the covariate is set to its sample mean, such that

$${\hat{\beta }}_{10}^{a_{0}}+{\hat{\beta }}_{11}^{a_{0}}\hat{E}(L_{0}|A_{0}=a_{0})=\frac{\sum_{i=1}^{n}I(A_{0,i}=a_{0})L_{1,i}}{\sum_{i=1}^{n}I(A_{0,i}=a_{0})}.$$

It follows that

$$\hat{E}(Y^{a_{1}=0}|A_{0}=a_{0})=\frac{\sum_{i=1}^{n}I(A_{0,i}=a_{0})\left({\hat{\beta }}_{20}^{a_{0}}+{\hat{\beta }}_{21}^{a_{0}}L_{0,i}+{\hat{\beta }}_{22}^{a_{0}}L_{1,i}\right)}{\sum_{i=1}^{n}I(A_{0,i}=a_{0})}$$

which is identical to the G-formula estimator given in Equation (11).

More commonly a linear mixed model is fitted which assumes a common covariance matrix for $\left(L_{1},Y^{a_{1}=0}\right)$ across the two randomized groups with mean effects of *A*_0_ and *L*_0_. A similar argument to the one above shows that the resulting estimator is a G-formula estimator where we fit a single model for $Y^{a_{1}=0}$ to both randomized groups, with *A*_0_ as a covariate (in addition to *L*_0_ and *L*_1_).

Finally, we note that a multiple imputation (MI) analysis assuming MAR and based on the same modeling assumptions as the likelihood based analysis is (up to Monte Carlo noise) equivalent to the likelihood based analysis. Thus, corresponding multiple imputation estimates which delete data after the ICE occurs are equivalent to particular G-formula implementations. The equivalence of imputation approaches and G-formula was previously discussed by Westreich et al. (2015) and Qu et al. (2020).

#### 3.2.2 Inverse Probability of Missing Weighting

The potential outcomes of interest among those randomized to *A*_0_ = *a*_0_ are $Y^{a_{1}=0}$ . Since the event that $A_{1}=0$ is precisely the indicator of observation of the potential outcome of interest, the standard IPW missing data estimator (Seaman and White 2013) for $E(Y^{a_{1}=0}|A_{0}=a_{0})$ can be seen to be identical to the “causal inference” IPW estimator given in Equation (12).

### 3.3 Deterministic Intercurrent Events

In some trials the intercurrent event could be discontinuation of randomized treatment or addition of rescue treatment. In some therapy areas, for example, diabetes, the protocol specifies that rescue treatment be given at/following a visit at time *k* if and only if a measurement of glycemic control (e.g., via FPG or HbA1c) exceeds some threshold. This has been termed a deterministic MAR situation (Holzhauer, Akacha, and Bermann 2015), with missing data estimation approaches advocated.

In such situations the positivity assumption is violated if the protocol was followed. This implies that the hypothetical estimand cannot be nonparametrically identified, which essentially means it cannot be estimated without making untestable modeling assumptions. Parametric G-formula, likelihood and MI approaches can provide consistent estimates, but only by extrapolating beyond the data. In particular, they must predict the no ICE potential outcomes for those individuals who did in fact have an ICE. When intercurrent events occur deterministically, there are no similar patients in terms of $A_{0},L_{0},L_{1}$ who did not have the ICE and hence have $Y^{a_{1}=0}$ observed. If the extrapolation implied by the model is correct, we can obtain consistent estimates. However, from the data alone, we have no basis on which to judge whether the extrapolation is justified. In such cases, the extrapolation can arguably only be justified on the basis of external evidence, since the data offer no information about whether the extrapolation is reliable.

In contrast, if the IPW approach is used, and the model for ICE/missingness correctly incorporates the deterministic ICE mechanism, for those with $A_{1}=0$ because $P(A_{1}=0|A_{0},L_{0},L_{1})=1$ their true weight will be 2 as per Equation (13), as well as for those with $A_{1}=1$ because $P(A_{1}=1|A_{0},L_{0},L_{1})=1$ . In this case, the estimator in Equation (12) would simply be the unweighted average of outcomes in those with $A_{1}=0$ , which in general will give a biased estimate.

## 4 Intercurrent Events at Multiple Time Points

**Fig. 5:**
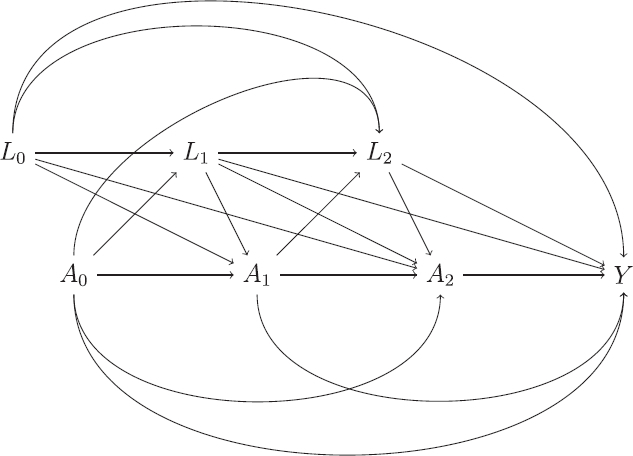
Directed acyclic graph (DAG) representation of randomized trial with ICE occurring at two time points.

We now consider the more realistic setting where the ICE can occur at multiple time points. We consider the case where the ICE can occur at two time points, denoted *A*_1_ and *A*_2_, and note that our conclusions in this situation can be easily extended to the general setup with more time points. The hypothetical potential outcomes of interest now are $Y^{a_{0},a_{1}=0,a_{2}=0}$ for $a_{0}=0$ and $a_{0}=1$ . Figure 5 shows the DAG for this situation.

### 4.1 Causal Inference Approaches

#### 4.1.1 G-formula

Using Equation (4) we have that

$$E(Y^{\bar{a}})=\int_{l_{0}}\int_{l_{1}}\int_{l_{2}}E(Y|\bar{a},\bar{l})f(l_{2}|{\bar{a}}_{1},{\bar{l}}_{1})f(l_{1}|a_{0},l_{0})f(l_{0})dl_{2}dl_{1}dl_{0}.$$

As before because of randomization $f(l_{0})=f(l_{0}|a_{0})$ and so we can write

(14) $$E(Y^{\bar{a}})=\int_{l_{0}}\int_{l_{1}}\int_{l_{2}}E(Y|\bar{a},\bar{l})f(l_{2}|{\bar{a}}_{1},{\bar{l}}_{1})f(l_{1},l_{0}|a_{0})dl_{2}dl_{1}dl_{0}.$$

To construct an estimator based on this, we specify and fit a model for $E(Y|\bar{A},\bar{L})$ and for $f(L_{2}|{\bar{A}}_{1},L_{0},L_{1})$ , while for $f(L_{1},L_{0}|A_{0}=a_{0})$ we can again avoid modeling it by using the empirical distribution of (*L*_1_, *L*_0_) in those randomized to treatment *a*_0_. As before, we could fit models for $E(Y|\bar{A},\bar{L})$ and $f(L_{2}|{\bar{A}}_{1},L_{0},L_{1})$ using data from all patients with suitable covariate effect specification, or instead restrict them to those whose treatment history $\bar{A}$ equals the hypothetical estimand regime of interest $\bar{a}=(a_{0},0,0)$ . The former may be more efficient, but it requires specification of more modeling assumptions. Taking the latter approach, suppose we assume that

(15) $$\begin{array}{c} E(Y|A_{0}=a_{0},A_{1}=0,A_{2}=0,L_{0},L_{1},L_{2})\\ \;\;\;=\beta_{30}^{a_{0}}+\beta_{31}^{a_{0}}L_{0}+\beta_{32}^{a_{0}}L_{1}+\beta_{33}^{a_{0}}L_{2}\\ E(L_{2}|A_{0}=a_{0},A_{1}=0,L_{0},L_{1})=\beta_{20}^{a_{0}}+\beta_{21}^{a_{0}}L_{0}+\beta_{22}^{a_{0}}L_{1}. \end{array}$$

Then we have that

$$\begin{array}{c} E\left\{E(Y|A_{0}=a_{0},A_{1}=0,A_{2}=0,L_{0},L_{1},L_{2})|A_{0}=a_{0},A_{1}=0,L_{0},L_{1}\right\}\\ \;=E\left\{\beta_{30}^{a_{0}}+\beta_{31}^{a_{0}}L_{0}+\beta_{32}^{a_{0}}L_{1}+\beta_{33}^{a_{0}}L_{2}|A_{0}=a_{0},A_{1}=0,L_{0},L_{1}\right\}\\ \;=\beta_{30}^{a_{0}}+\beta_{31}^{a_{0}}L_{0}+\beta_{32}^{a_{0}}L_{1}+\beta_{33}^{a_{0}}E(L_{2}|A_{0}=a_{0},A_{1}=0,L_{0},L_{1})\\ \;=\beta_{30}^{a_{0}}+\beta_{31}^{a_{0}}L_{0}+\beta_{32}^{a_{0}}L_{1}+\beta_{33}^{a_{0}}(\beta_{20}^{a_{0}}+\beta_{21}^{a_{0}}L_{0}+\beta_{22}^{a_{0}}L_{1})\\ \;=\beta_{30}^{a_{0}}+\beta_{33}^{a_{0}}\beta_{20}^{a_{0}}+(\beta_{31}^{a_{0}}+\beta_{33}^{a_{0}}\beta_{21}^{a_{0}})L_{0}+(\beta_{32}^{a_{0}}+\beta_{33}^{a_{0}}\beta_{22}^{a_{0}})L_{1}. \end{array}$$

Then using Equation (14) our G-formula estimator is

(16) $$\frac{\begin{array}{c} \sum_{i=1}^{n}I(A_{0,i}=a_{0})\{{\hat{\beta }}_{30}^{a_{0}}+{\hat{\beta }}_{33}^{a_{0}}{\hat{\beta }}_{20}^{a_{0}}+({\hat{\beta }}_{31}^{a_{0}}+{\hat{\beta }}_{33}^{a_{0}}{\hat{\beta }}_{21}^{a_{0}})L_{0,i}\\ +({\hat{\beta }}_{32}^{a_{0}}+{\hat{\beta }}_{33}^{a_{0}}{\hat{\beta }}_{22}^{a_{0}})L_{1,i}\} \end{array}}{\sum_{i=1}^{n}I(A_{0,i}=a_{0})}.$$

In words, for each patient randomized to treatment *a*_0_, this G-formula estimator first predicts $L_{2}^{a_{0},a_{1}=0}$ under the hypothetical no ICE scenario and then predicts $Y^{a_{0},a_{1}=0,a_{2}=0}$ . Finally, it averages these predictions across the patients randomized to *a*_0_.

#### 4.1.2 Inverse Probability of Treatment Weighting

For this setting when the ICE can occur at two time points, the IPW estimator for the mean of $Y^{a_{0},a_{1}=0,a_{2}=0}$ is a weighted mean of the outcomes from those patients who were randomized to treatment *a*_0_ and in whom the ICE did not occur at either of the two intermediate time points. The weights are as defined in Equation (6).

Unlike in the setting considered in Section 3, we could now choose to model the occurrence of ICE at each time point using all patients, with earlier occurrence of ICE as a covariate. However, since in the end we only need weights for those patients who did not experience the ICE at either time point, we might choose instead to model the occurrence of the ICE at time *k* only among those who had not up to time *k* experienced an ICE. Thus, like the G-formula, one has some flexibility and choice about which data to use and what modeling assumptions to make. Indeed, one may wish to avoid modeling how the occurrence of an ICE depends on the past among those who have already experienced an ICE.

### 4.2 Missing Data Approaches

#### 4.2.1 Likelihood Based Missing Data Approaches

As noted earlier, missing data methods have previously been advocated to and applied for estimating hypothetical estimands by fitting mixed models to the repeated measurements of outcomes after excluding any post ICE outcomes. This leads to a so called monotone missingness pattern. We now show using a SWIG derived from the DAG in Figure 5 that the hypothetical potential outcomes of interest are again MAR.

The full data under the hypothetical estimand is now $\left(L_{1},L_{2}^{a_{1}=0},Y^{a_{1}=0,a_{2}=0}\right)$ . There are missing values in $L_{2}^{a_{1}=0}$ and $Y^{a_{1}=0,a_{2}=0}$ , and if $L_{2}^{a_{1}=0}$ is missing for an individual then so is $Y^{a_{1}=0,a_{2}=0}$ . This is analogous to monotone dropout in a longitudinal study. In this context, MAR can be expressed as saying that at any given time, among those subjects who have not yet dropped out, the probability of dropout before the next follow-up visit is independent of future outcomes given the past outcomes (Daniels and Hogan 2008).

**Fig. 6:**
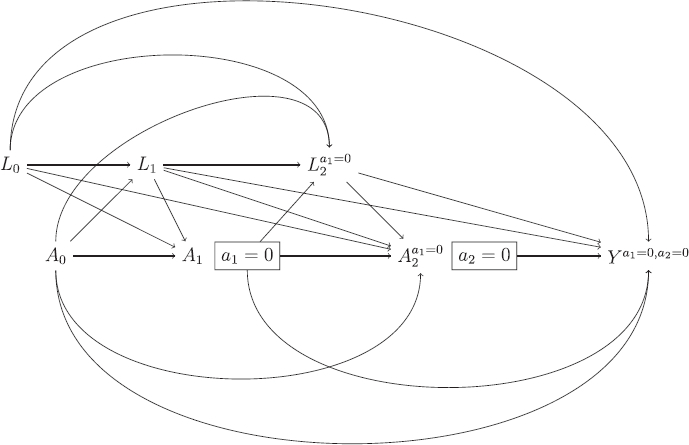
SWIG resulting from DAG shown in Figure 5, intervening to set $a_{1}=0$ and $a_{2}=0$ .

Figure 6 shows the SWIG resulting from the DAG in Figure 5 if we intervene to set $a_{1}=0$ and $a_{2}=0$ , and we can use this to check MAR is satisfied. First we can immediately confirm from the SWIG that $A_{1}⊥⊥(L_{2}^{a_{1}=0},Y^{a_{1}=0,a_{2}=0})|A_{0},L_{1},L_{0}$ . Next we must check that $A_{2}⊥⊥Y^{a_{1}=0,a_{2}=0}|A_{1}=0,A_{0},L_{2},L_{1},L_{0}$ . For this, note that in those with $A_{1}=0$ , by the consistency assumption $L_{2}=L_{2}^{a_{1}=0}$ and $A_{2}=A_{2}^{a_{1}=0}$ , and so this assumption is equivalent to $A_{2}^{a_{1}=0}⊥⊥Y^{a_{1}=0,a_{2}=0}|A_{1}=0,A_{0},L_{2}^{a_{1}=0},L_{1},L_{0}$ , and the SWIG shows that this conditional independence condition indeed holds. We emphasize again, that if there are, as there typically would be, common causes of ICE occurrence and final outcome additional to the repeated measurements of outcome, they must be included in *L*_0_, *L*_1_, *L*_2_. Others have previously discussed the use of missing data methods assuming MAR whereby data after the ICE occurs are excluded from the analysis (Holzhauer, Akacha, and Bermann 2015; Mallinckrodt et al. 2020). By using the machinery of causal diagrams, we are able to clarify the conditions under which the MAR assumption would be satisfied—namely that we have measured and conditioned on all common causes of the ICE variables (here *A*_1_ and *A*_2_) and the final outcome of interest (here *Y*).

As was the case with an ICE occurring at a single time point, in the supplementary material we show that particular “missing data estimators” and particular “causal inference estimators” are equivalent. The fact that missing data approaches are valid and are identical to particular causal inference estimators in this setting is perhaps at first sight surprising, since it is often said that a setting with time-dependent confounding affected by treatment is one in which “standard approaches” are invalid, with more complex g-methods required instead. For further details, see the supplementary materials.

#### 4.2.2 Inverse Probability of Missing Weighting

As before we note that we are considering the ICE as a “treatment” and it is also the missing indicator because we are only interested in the potential outcomes $Y^{a_{1}=0,a_{2}=0}$ which would occur in the absence of the ICE. However, when the ICE can occur at more than one time point, the implementation of IPW for missingness is equivalent to the one described in Section 4.1.2, where we fit the models to estimate the weights at time point *k* restricted to those with ${\bar{A}}_{1:k-1}=\bar{0}$ .

## 5 Conclusion

To target a hypothetical estimand under no ICE, we have considered different estimators arising from the causal inference and missing data literature. In doing so, we have shown that there are close connections between estimators in these groups. Indeed, we have shown that “missing data” likelihood based estimators (observed data likelihood or multiple imputation) applied after data post-ICE are deleted are implementations of the G-formula method from causal inference. Similarly, inverse probability of missing estimators for hypothetical estimands are also inverse probability of treatment estimators from causal inference.

We believe this unification is helpful to those analyzing clinical trial data not least because the assumptions required for estimation, expressed via causal inference language, are arguably more easily understood than missing data assumptions. In particular, we believe DAGs can be very useful tools for graphically encoding what we may view as plausible for the causal relationships between variables, and from this, the validity of the sequential exchangeability assumption can be assessed.

The causal inference lens also brings to the fore the importance of the positivity assumption. In trials where ICE occurrence is a deterministic function of biomarker values, such that the assumption is violated, estimation via likelihood methods or G-formula relies on extrapolation. The reasonableness of this extrapolation should be assessed on a case by case basis, taking into account the type of ICE, the disease context, and the extent of the extrapolation being made. Sensitivity analyses may be required given that the reliability of the extrapolation cannot be assessed from the observed data. Alternatively, in such cases one may choose to target a different estimand (Michiels et al. 2021).

It is worth noting that, in a given context, different hypothetical estimands can be defined for a particular ICE. For instance, in the case of use of rescue medication, we could conceive a hypothetical scenario where rescue medication was not available for the early stage of the trial that is, no rescue medication in the first 6 months in a 36 months follow-up trial. The alternatives could be to set a shorter/longer period of follow-up or to consider not allowing rescue medication use in the protocol. The hypothetical strategy could be regarded even more broadly and instead of considering an intervention to set the ICE to 0 for everyone, this could be set to a different value. For example, this could mean assessing the randomized treatment versus control treatment where everyone was to receive rescue medication.

While we have shown that commonly used “missing data estimators” for hypothetical estimands correspond to certain causal inference estimators, we have also seen that there are additional implementations of G-formula and IPW which could be used instead. For example, contrary to current common practice, it is possible to use the full data, including intermediate and final outcomes assessed after the occurrence of the ICE, so long as suitable adjustment is made for past ICE occurrence. As seen in the simulations (supplementary material), this approach offers the potential for more precise estimates, at the expense of relying on more modeling assumptions.

There can be settings where it is plausible to borrow information from patients with ICEs. Consider a diabetes trial assessing the impact of a novel treatment with standard of care on achieving normal HbA1c levels. If a patient discontinues the novel treatment and still achieves normal HbA1c levels, they would probably have also had a positive outcome had they continued on treatment.

It is worth noting that the hypothetical estimand is particularly relevant to deal with ICEs that can be intervened on. For instance we could conceive a trial where we could intervene to avoid treatment interruptions due to administrative reasons such as the ones derived from government enforced closures during the current COVID-19 pandemic. In contrast, it would probably be less sensible to consider a world were we could intervene to avoid treatment discontinuation due to adverse events. For these cases, a different strategy to deal with the ICE is more reasonable.

We have assumed there is only one ICE under consideration. In practice there is typically more than one ICE. With multiple types of ICE which are all chosen to be handled using the hypothetical strategy, the methods described here could be applied with the $A_{1},\ldots ,A_{K}$ now denoting occurrence of at least one of the ICEs. However, in order to avoid model misspecification it may be preferable to define *A_k_* as a vector indicating occurrence or not of each of the ICE types at time *k*. If some ICE are to be handle with using the hypothetical strategy and some using treatment policy, if the treatment policy ICE precedes the hypothetical ICE in time, it may be possible to consider the treatment policy ICE as an additional time-varying covariate (i.e., as part of $L_{1},\ldots ,L_{K}$ ). We will address in more detail estimation in the case of two or more ICE types in a subsequent paper.

The validity of the estimates given by the methods described depends on the models being correctly specified, as shown by the simulations (supplementary material). To make the estimates more robust to model misspecification, so-called doubly-robust estimators were developed (Bang and Robins 2005). The idea is that the estimates are derived using two separate models and only one of the models needs to be correctly specified to obtain consistent estimates. The first model typically concerns the treatment assignment (propensity score model) while the second model is a model for the outcome. Section 21.3 of Hernan and Robins (2020) describes calculation of a doubly robust estimator in the time-varying treatment setting, which could be applied for estimation of hypothetical estimands. To provide further robustness to model misspecification, machine learning approaches could also be explored (Van Der Laan and Rubin 2006).

For the methods covered in the article, we did not discuss variance estimation. First, the purpose of the article was to provide feasible ways to implement existing estimators to target hypothetical estimands, so the main focus was on the treatment effect estimate. More importantly, as these are well established estimators, there is already existing literature proposing different ways of estimating the corresponding variance, including bootstrapping and sandwich estimators. In fact, there are different packages available to implement them in standard software (van der Wal and Geskus 2011; McGrath et al. 2020).

We also did not discuss G-estimation (Hernan and Robins 2020). The difference between G-estimation and the other G-methods is that G-estimation estimates conditional treatment effects within levels of *L*. In settings where conditional causal effects are of interest, G-estimation may be of relevance.

In practice, there may be data missing for example, due to missed visits. As we have discussed, certain estimators do not use data after the occurrence of an ICE, and so missingness in variables at follow-up visits occurring after ICEs present no difficulties for these estimators. For patients with missing values before occurrence of the ICE, their missing hypothetical (no ICE) outcomes could be imputed, for example using MI. There would be no need to differentiate between such missing values and dataset missing because of the ICE, provided both common causes of missingness and outcome, and common causes of the ICE and outcome are conditioned on in the imputation models. Alternatively, MI could first be used to impute any missing actual (as opposed to hypothetical counterfactuals) data, following which any of the methods described previously (e.g., G-formula or IPW) could be applied to estimate the hypothetical no ICE estimand. Dealing with missing actual data with IPW is less attractive, since its implementation when missingness is non-monotone (as it often is), is difficult (Sun and Tchetgen Tchetgen 2018). Moreover, use of IPW may require one to specify separate models for the missingness and occurrence of ICE processes.

We hope that by drawing parallels between causal inference and missing data methods and describing how the different estimators work, the assumptions required for valid estimates and showing feasible ways to implement them, researchers involved in the design and analysis of clinical trials will be able to successfully apply these methods to their trials.

## Supplementary Material

Supplemental Material

## Supplementary Materials

Equivalence of G-formula and likelihood based missing data approaches - Simulations.
