# Supplementary material for: Hypothetical Estimands in Clinical Trials: A Unification of Causal Inference and Missing Data Methods
Source: Stat Biopharm Res. 2022 Jul 6;15(2):421–32. doi: 10.1080/19466315.2022.2081599 (PMC10228513; doi:10.1080/19466315.2022.2081599)
Supplement: Supplemental Material [file USBR_A_2081599_SM4742.pdf]

SUPPLEMENTARY MATERIAL

Equivalence of G-formula and likelihood based missing data approaches

Following Figure 6, consider the data on (L_0, L_1, L_2, Y) in those with $A_0 = a_0$ resulting after deletion of the L_2 and Y values for individuals with $A_1 = 1$ and deletion of the Y values for those with $A_1 = 0$ and $A_2 = 1$. Suppose we assume the 'full data' on $(L_1, L_2^{a_1=0}, Y^{a_1=0,a_2=0})$ are tri-variate normal, with their means depending on L_0 linearly with distinct coefficients and an unstructured covariance matrix. Then viewing $(L_1, L_2^{a_1=0}, Y^{a_1=0,a_2=0})$ as repeated measures, as per the case in Section 3, this implies that the conditional means of each 'outcome' given the earlier values (and L_0) are

$$\begin{aligned} E(Y^{a_1=0,a_2=0}|A_0 = a_0, L_0, L_1, L_2^{a_1=0}) &= \beta_{30}^{a_0} + \beta_{31}^{a_0} L_0 + \beta_{32}^{a_0} L_1 + \beta_{33}^{a_0} L_2^{a_1=0} \\ E(L_2^{a_1=0}|A_0 = a_0, L_0, L_1) &= \beta_{20}^{a_0} + \beta_{21}^{a_0} L_0 + \beta_{22}^{a_0} L_1 \\ E(L_1|A_0 = a_0, L_0) &= \beta_{10}^{a_0} + \beta_{11}^{a_0} L_0 \end{aligned}$$

Moreover, under MAR the observed data MLEs of the parameters in these models are obtained by fitting the model for $Y^{a_1=0,a_2=0}$ in those with $A_1 = 0$ and $A_2 = 0$, the model for $L_2^{a_1=0}$ in those with $A_1 = 0$ and the model for L_1 using all patients with $A_0 = a_0$. Then we have that

$$\begin{aligned} E(Y^{a_1=0,a_2=0}|A_0 = a_0, L_0, L_1) &= \beta_{30}^{a_0} + \beta_{31}^{a_0} L_0 + \beta_{32}^{a_0} L_1 + \beta_{33}^{a_0}(\beta_{20}^{a_0} + \beta_{21}^{a_0} L_0 + \beta_{22}^{a_0} L_1) \\ E(Y^{a_1=0,a_2=0}|A_0 = a_0, L_0) &= \beta_{30}^{a_0} + \beta_{31}^{a_0} L_0 + \beta_{32}^{a_0}(\beta_{10}^{a_0} + \beta_{11}^{a_0} L_0) + \beta_{33}^{a_0}(\beta_{20}^{a_0} + \beta_{21}^{a_0} L_0 + \beta_{22}^{a_0}(\beta_{10}^{a_0} + \beta_{11}^{a_0} L_0)) \\ E(Y^{a_1=0,a_2=0}|A_0 = a_0) &= \beta_{30}^{a_0} + \beta_{31}^{a_0} E(L_0|A_0 = a_0) + \beta_{32}^{a_0}(\beta_{10}^{a_0} + \beta_{11}^{a_0} E(L_0|A_0 = a_0)) \\ &\quad + \beta_{33}^{a_0}(\beta_{20}^{a_0} + \beta_{21}^{a_0} E(L_0|A_0 = a_0) + \beta_{22}^{a_0}(\beta_{10}^{a_0} + \beta_{11}^{a_0} E(L_0|A_0 = a_0))) \end{aligned}$$

As in Section 3, the non-parametric MLE of $E(L_0|A_0 = a_0)$ is $\hat{E}(L_0|A_0 = a_0) = \frac{\sum_{i=1}^n I(A_{0,i}=a_0)L_{0,i}}{\sum_{i=1}^n I(A_{0,i}=a_0)}$. Then the MLE of $E(Y^{a_1=0,a_2=0}|A_0 = a_0)$ is

$$\begin{aligned} \hat{E}(Y^{a_1=0,a_2=0}|A_0 = a_0) &= \hat{\beta}_{30}^{a_0} + \hat{\beta}_{31}^{a_0} \hat{E}(L_0|A_0 = a_0) + \hat{\beta}_{32}^{a_0}(\hat{\beta}_{10}^{a_0} + \hat{\beta}_{11}^{a_0} \hat{E}(L_0|A_0 = a_0)) \\ &\quad + \hat{\beta}_{33}^{a_0}(\hat{\beta}_{20}^{a_0} + \hat{\beta}_{21}^{a_0} \hat{E}(L_0|A_0 = a_0) + \hat{\beta}_{22}^{a_0}(\hat{\beta}_{10}^{a_0} + \hat{\beta}_{11}^{a_0} \hat{E}(L_0|A_0 = a_0))) \\ &= \hat{\beta}_{30}^{a_0} + \hat{\beta}_{31}^{a_0} \hat{E}(L_0|A_0 = a_0) + \hat{\beta}_{32}^{a_0} \hat{E}(L_1|A_0 = a_0) \\ &\quad + \hat{\beta}_{33}^{a_0}(\hat{\beta}_{20}^{a_0} + \hat{\beta}_{21}^{a_0} \hat{E}(L_0|A_0 = a_0) + \hat{\beta}_{22}^{a_0} \hat{E}(L_1|A_0 = a_0)) \\ &= \frac{\sum_{i=1}^n I(A_{0,i} = a_0)\{\hat{\beta}_{30}^{a_0} + \hat{\beta}_{33}^{a_0}\hat{\beta}_{20}^{a_0} + (\hat{\beta}_{31}^{a_0} + \hat{\beta}_{33}^{a_0}\hat{\beta}_{21}^{a_0})L_{0,i} + (\hat{\beta}_{32}^{a_0} + \hat{\beta}_{33}^{a_0}\hat{\beta}_{22}^{a_0})L_{1,i}\}}{\sum_{i=1}^n I(A_{0,i} = a_0)} \end{aligned}$$

which is the G-formula estimator of equation 16.

The key difference between the g-formula and standard regression in settings with time-varying treatment such as the one outlined in this paper is that g-formula in general fits models sequentially, to each L_k $(k > 0)$ and Y given the past, and effectively predicts (or

more generally simulates) each time-dependent confounder, $\tilde{L}_k^{a_0,\ldots,a_{k-1}}$, under each treatment regime to be compared. When estimating the mean potential outcome at the final time-point under a given regime, the predictions for Y that are averaged are based on the fitted model for Y given the past, but with the treatments set to their values under the regime, and the confounders set to their predicted (or more generally simulated) values, again under the relevant regime. For example, if we write $m(a_0, a_1, a_2, l_0, l_1, l_2)$ for the estimated prediction function corresponding to $E(Y|a_0, a_1, a_2, l_0, l_1, l_2,)$ then $E(Y^{a_0=a_1=a_2=0})$ would be estimated as

$$\frac{1}{n}\sum_{i=1}^{n} m(0, 0, 0, L_{0,i}, \tilde{L}_{1,i}^{a_0=0}, \tilde{L}_{2,i}^{a_0=a_1=0})$$

but $E(Y^{a_0=1,a_1=a_2=0})$ would be estimated as

$$\frac{1}{n}\sum_{i=1}^{n} m(1, 0, 0, L_{0,i}, \tilde{L}_{1,i}^{a_0=1}, \tilde{L}_{2,i}^{a_0=1,a_1=0}).$$

Crucial to the success of the above strategy for correctly allowing for time-dependent confounding affected by treatment is that, in general, $\tilde{L}_{1,i}^{a_1=0} \neq \tilde{L}_{1,i}^{a_1=1}$, and so on.

How is it possible, therefore, for standard missing data methods to be equivalent to g-formula in the setting considered in this paper, even though the missing data approach appears to perform the estimation (which can usefully for the purposes of this paragraph be thought of as an imputation-based procedure) under *only one* regime? There are three components to the answer to this question. First of all, we are only interested in two regimes, namely $(1, 0, 0)$ and $(0, 0, 0)$: active versus control with all ICEs prevented. Second, the initial treatment is randomised (there is no confounding by L_0), and thus, instead of needing to simulate L_1, L_2 and Y based on L_0 for *all* participants under the two regimes $(1, 0, 0)$ and $(0, 0, 0)$ before standardising to the overall distribution of L_0, we can simulate L_1, L_2 and Y once for each participant under the regime $(A_0, 0, 0)$ and take the average within each treatment arm separately as estimates of $E(Y^{1,0,0})$ and $E(Y^{0,0,0})$. In an observational study, such a strategy would be biased due to the unaccounted confounding by L_0, but in an (initially) randomised trial, imputing separately for each arm of the trial, and thus, only under one regime ('assigned treatment followed by no ICE') for each participant is valid. The third reason why the missing data approaches work is that they delete data once an ICE has occurred, so that all the remaining data are observed under the regime of interest, and hence the implied imputations (under an MAR assumption) for all the deleted data are also made under the regime of interest, exactly as would happen in the g-formula.

The previous paragraph highlights an important consideration when using missing data methods to estimate the hypothetical estimand, namely that it is not only previous measurements of the outcome that need to be deleted in those for whom an ICE has occurred, but any other time-varying common cause of A_k and Y that could plausibly be affected by treatment. In other words, any L_k must be treated in the same way as Y, irrespective of whether or not it is an intermediate measurement of the final outcome of interest.

Simulations

To illustrate and compare the different estimation methods' performance, we conducted simulations with probabilistic or deterministic ICE occurrence. We also explore the impacts of misspecification in the models used by the different estimation approaches. In what follows, $\bar{c}$ denotes a column vector of suitable length for conformability with all elements equal to the scalar c.

Probabilistic intercurrent event

We generated $10,000$ datasets of 500 subjects where the treatment A_0 was assigned 1:1 at random and the ICE could occur at 5 different time points during follow-up ($K = 5$) as follows:

- $A_0 \sim Ber(0.5)$
- $L_0 \sim \mathcal{N}(0, 1)$
- $L_k \sim \mathcal{N}(\overline{0.3}^T \bar{L}_{k-1} + \overline{0.2}^T \bar{A}_{k-1}, 1)$ for $k = 1 : 5$
- $A_k \sim Ber\Big(\text{expit}(-3 + \overline{0.2}^T \bar{L}_k + \overline{0.4}^T \bar{A}_{k-1})\Big)$ for $k = 1 : 5$
- $Y \sim \mathcal{N}(\overline{0.2}^T \bar{L} + 0.5A_0 + \overline{0.3}^T \bar{A}_{1:5}, 1)$

The parameter values were chosen so that on average 60-70 % would be ICE free at the end of follow-up, for the different scenarios considered.

Each of the generated datasets was analysed using the following methods:

1. Naive: treatment effect estimated as the difference in mean outcome between the randomised treatment arms, among those who did not experience the ICE:

$$\widehat{E}(Y|A_0 = 1, \bar{A}_{1:5} = \bar{0}) - \widehat{E}(Y|A_0 = 0, \bar{A}_{1:5} = \bar{0})$$
$$= \frac{\sum_{i=1}^n I(A_i = (1, \bar{0}))Y_i}{\sum_{i=1}^n I(A_i = (1, \bar{0}))} - \frac{\sum_{i=1}^n I(\bar{A}_i = (0, \bar{0}))Y_i}{\sum_{i=1}^n I(\bar{A}_i = (0, \bar{0}))}$$

2. G-formula using all data:

   (a) First fit linear models for L at each time point $k \in 1 : 5$, given the observed covariate history until time k $(\bar{A}_{k-1}, \bar{L}_{k-1})$, including everyone, regardless of whether they had the ICE at anytime during follow-up or not: $\widehat{E}(L_k|\bar{A}_{k-1}, \bar{L}_{k-1})$, with main effects of each past A variable and each past L variable.

   (b) Linear model for $\widehat{E}(Y|\bar{A}, \bar{L})$ is fitted including everyone, with main effects of each A variable and each L variable.

(c) For every individual, a value of $\widehat{L}_{k,i}$ is predicted from the model at each time point, given their randomised treatment $A_{0,i}$, observed baseline covariate $L_{0,i}$, predicted covariate history up until that time point k ($\widehat{\bar{L}}_{k-1,i}$), and setting $\bar{A}_{1:k} = 0$.

(d) For every individual, a value of $\widehat{Y}_i$ is predicted from the outcome model, given their randomised treatment $A_{0,i}$, observed baseline covariate $L_{0,i}$, full predicted covariate history $\widehat{\bar{L}}$, and setting $\bar{A}_{1:5} = 0$.

(e) The treatment effect estimate is the difference in the means of $\widehat{Y}_i$ between the $A_0 = 1$ and $A_0 = 0$ groups, i.e. $\hat{E}(\widehat{Y}|A_0 = 1) - \hat{E}(\widehat{Y}|A_0 = 0)$.

3. G-formula among ICE-free: similar to the previous one but the linear models for L_ks and Y are fitted only including subjects ICE-free up until the corresponding time point.

4. G-formula separately by treatment arm: similar to the first G-formula implementation but the linear models for Ls and Y are fitted separately by randomised treatment groups.

5. G-formula among ICE-free separately by treatment arm: similar to the previous one where the linear models for Ls and Y are fitted separately by randomised treatment groups but among those ICE-free up until the corresponding time point.

6. Inverse probability of ICE weighting:

   (a) First a logistic regression is fitted to estimate the probability of the occurrence of the ICE at each time point, given the randomised treatment, observed ICE history, and observed covariate history: $P(A_k = 1|\bar{A}_{k-1}, \bar{L}_k)$ with main effects of each past A variable and each past L variable.

   (b) The weights W_i are calculated according to equation 6.

   (c) The treatment effect estimate is calculated using equation 7.

7. Inverse probability of ICE weighting among ICE-free: this is similar to the previous method but the difference is that the logistic regression at each time point k is fitted including only those ICE free until that time point ($\bar{A}_{1:k-1} = \bar{0}$), and there is no adjustment for past As (which are all zero in this subset).

8. Separate inverse probability of ICE weighting per arm treatment: similar to the first IPW version but fitting weight models separately by treatment arm.

9. Separate inverse probability of ICE weighting per arm treatment among ICE-free: similar to the previous version where the weight models are fitted separately by treatment arm but among those ICE-free up until the corresponding time point.

10. Multiple imputation with treatment as covariate using the `mice` package:

    (a) First, all the observations (Ls and Y) that occur after the first ICE are deleted.

(b) Sequential imputation of each L is performed using normal linear regression, adjusting for main effects of A_0 and past Ls.

(c) Imputation of Y using normal linear regression, adjusting for A_0 and past Ls.

(d) The treatment effect estimate was the difference in mean of Y between $A_0 = 1$ and $A_0 = 0$ groups across the 10 imputed datasets.

11. Multiple imputation separately by treatment arm: similar to the previous one but the imputation models are constructed separately for each treatment arm.

The results of the simulations are presented as box-and-whisker plots. For convenience, the extreme values were removed. Thus, any values larger than 1.5 times the interquartile range below or above the 25th percentile (lower hinge) and 75th percentile (upper hinge) respectively are not shown. The R code used for this simulations together with the plots containing the extreme values are freely available in the GitHub repository (URL: `https://github.com/colartep/hypothetical_estimands`).

Figure 1 shows the comparison of the different estimators to estimate the mean outcome under treatment, control and their difference i.e. treatment effect, under no ICE. The naive estimator (Method 1) was biased for the potential outcome means and their contrast, as expected.

Since all models used were correctly specified, the different implementations of the G-formula (Methods 2-5), IPW (Methods 6-9) and multiple imputation (Methods 10-11) provided unbiased estimates. From the different G-formula versions, it is evident that when using all the available information the precision is improved, as expected. For G-formula (Methods 4-5), IPW (Methods 6-9) and MI (Methods 11), fitting separate models by randomised arm led to more variability in estimates. As anticipated, the IPW estimators (Methods 6-9) were more variable than the G-formula estimators (Methods 2-5).

As discussed in Section 3.2.1, certain G-formula implementations and MI estimators are equivalent (up to Monte Carlo error). Here, Methods 3 and 10 are equivalent as well as Method 5 and 11. If desired, the Monte Carlo error can be reduced by using a larger number of imputations.

Deterministic switching

The initial data generating mechanism was modified to generate an ICE which will only occur if the value of the time-varying covariate in that visit exceeds a threshold as follows:

$$A_k = \begin{cases} 1 & \text{if } L_k \geq 1.5 \\ 0 & \text{otherwise} \end{cases} \quad \text{for } k = 1:5 \tag{1}$$

Figure 2 shows the results under this deterministic setting. As described in Section 3.3, when the ICE is deterministic given the covariates, the weights are the same for everyone and the IPW is equivalent to an unweighted average among those ICE free which corresponds to the naive estimator here. Thus, we do not include the IPW estimators for this setting. The G-formula and MI estimators give unbiased results because the extrapolation beyond the observed data they make is based on correctly specified models.

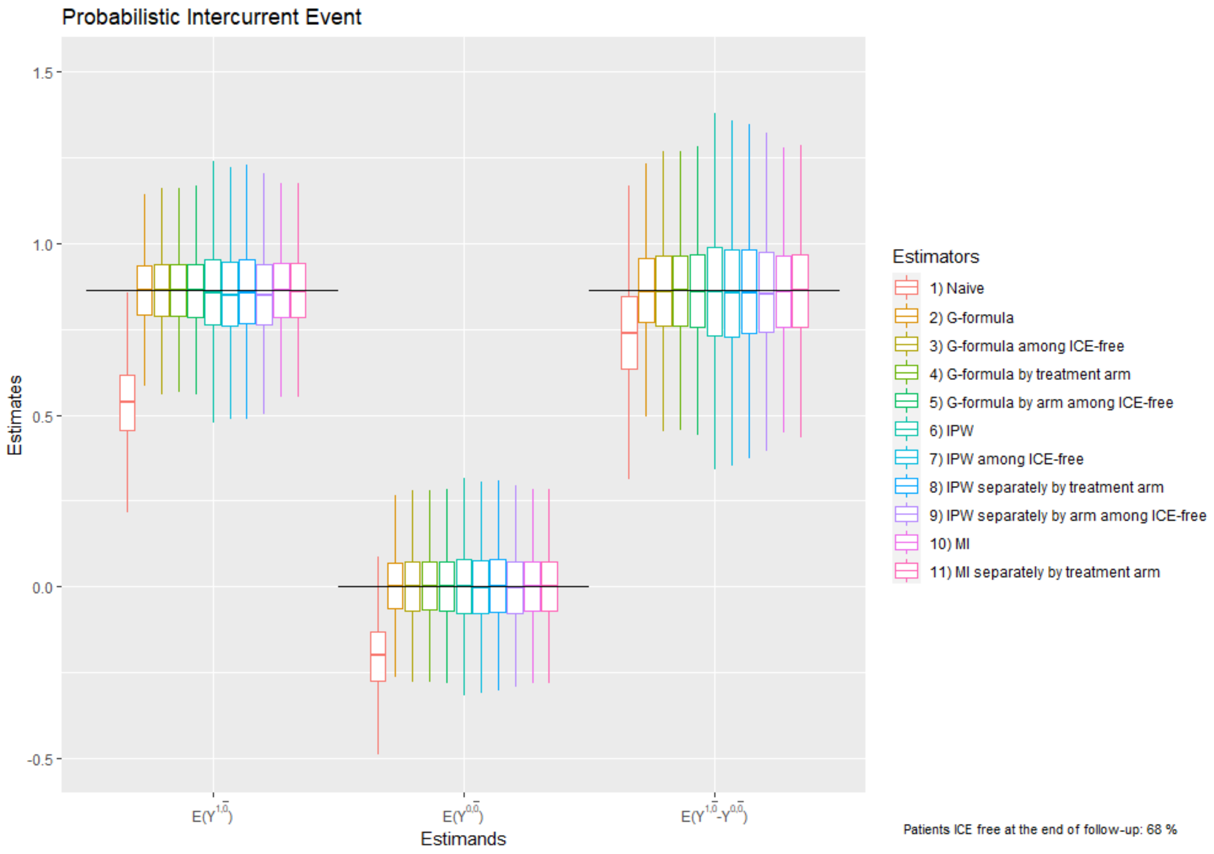

Figure 1: Simulation results showing estimates of potential outcome means under no ICE for $A_0 = 0$ and $A_0 = 1$ and their contrast, by different methods.

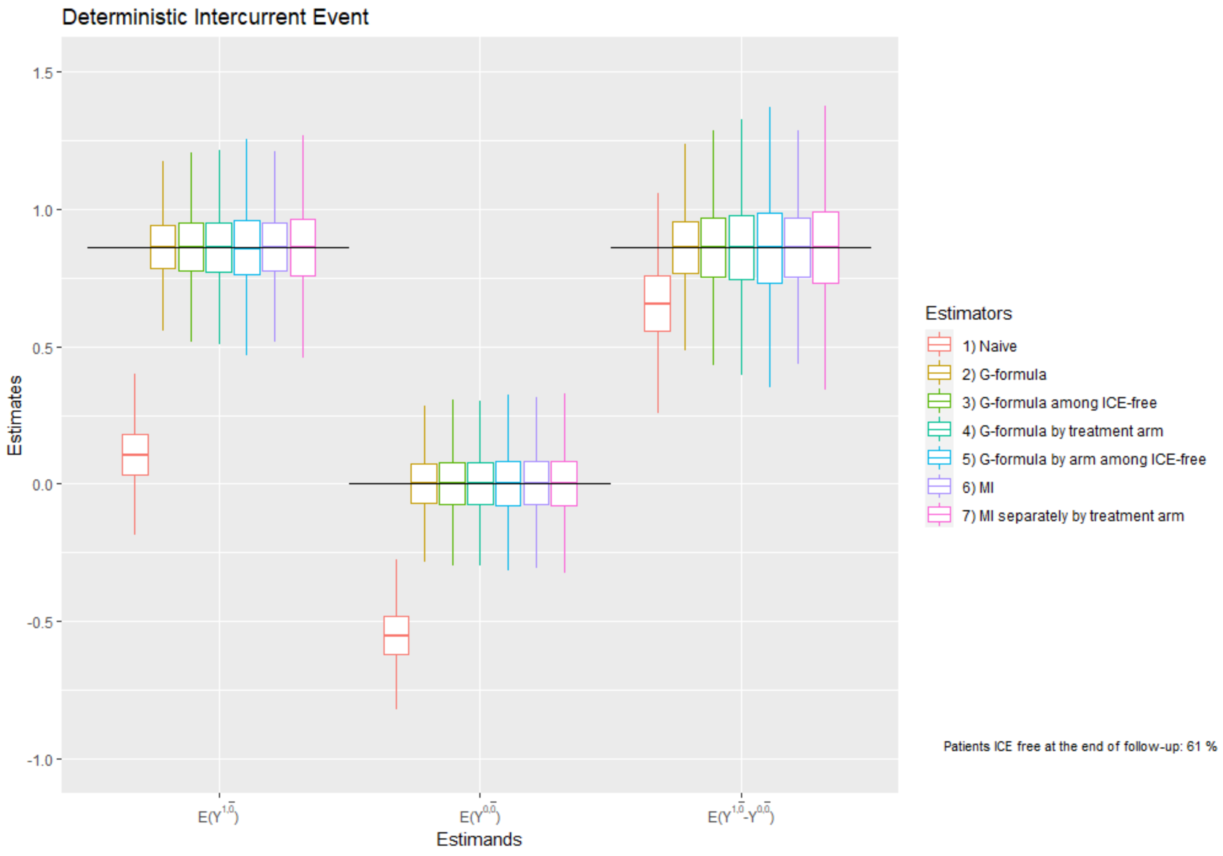

Figure 2: Simulation results showing the treatment effect as estimated by different methods, when the intercurrent event is determined non-randomly by the value of the time-varying covariates L.

Model misspecification

To investigate the impacts of model misspecification, we introduce an additional term, namely ${L_0}^2$ to the Y model in the data generating process. We then assess the impact on the estimation of the treatment effect without adapting the estimators previously described. We also introduced the quadratic term to the L_K model and A_K model in turn to assess their respective impact. Finally we considered a setting where all the models had this additional term.

Of note, if the direction of the bias is the same when estimating the two treatment regimens of interest, the bias may approximately cancel out for the treatment effect estimate. Thus, we allowed for differential magnitude and direction of the bias between treatment arms as follows:

- $L_5 \sim \mathcal{N}(\overline{0.3}^T \bar{L}_4 + \overline{0.2}^T \bar{A}_4 + 2{L_0}^2 A_0 - 0.5{L_0}^2(1 - A_0), 1)$ for $k = 1:5$
- $A_5 \sim Ber\Big(\text{expit}(-3 + \overline{0.2}^T \bar{L}_k + \overline{0.4}^T \bar{A}_{k-1} + 2{L_0}^2 A_0 - 0.5{L_0}^2(1 - A_0))\Big)$ for $k = 1:5$
- $Y \sim \mathcal{N}(\overline{0.2}^T \bar{L} + 0.5A_0 + \overline{0.3}^T \bar{A}_{1:5} + 2{L_0}^2 A_0 - 0.5{L_0}^2(1 - A_0), 1)$

Figure 3 presents the results when the outcome model is misspecified. Here the different implementations of the G-formula (Methods 2-5) and multiple imputation (Methods 10-11) give biased results as expected. As IPW does not rely on modelling the outcome (Methods 6-9), it is robust in this situation and provides unbiased results, although it is dramatically more variable. A similar situation is observed when the L-model is misspecified as shown in Figure 4.

The opposite situation is encountered when the ICE models are misspecified, as shown in Figure 5. Here, the IPW estimators (Methods 6-9) give biased results, while G-formula (Methods 2-5) and MI (Methods 10-11) are not because the latter do not depend on modelling the ICE occurrence. When all models are misspecified, the different estimators considered fail to give unbiased estimates.

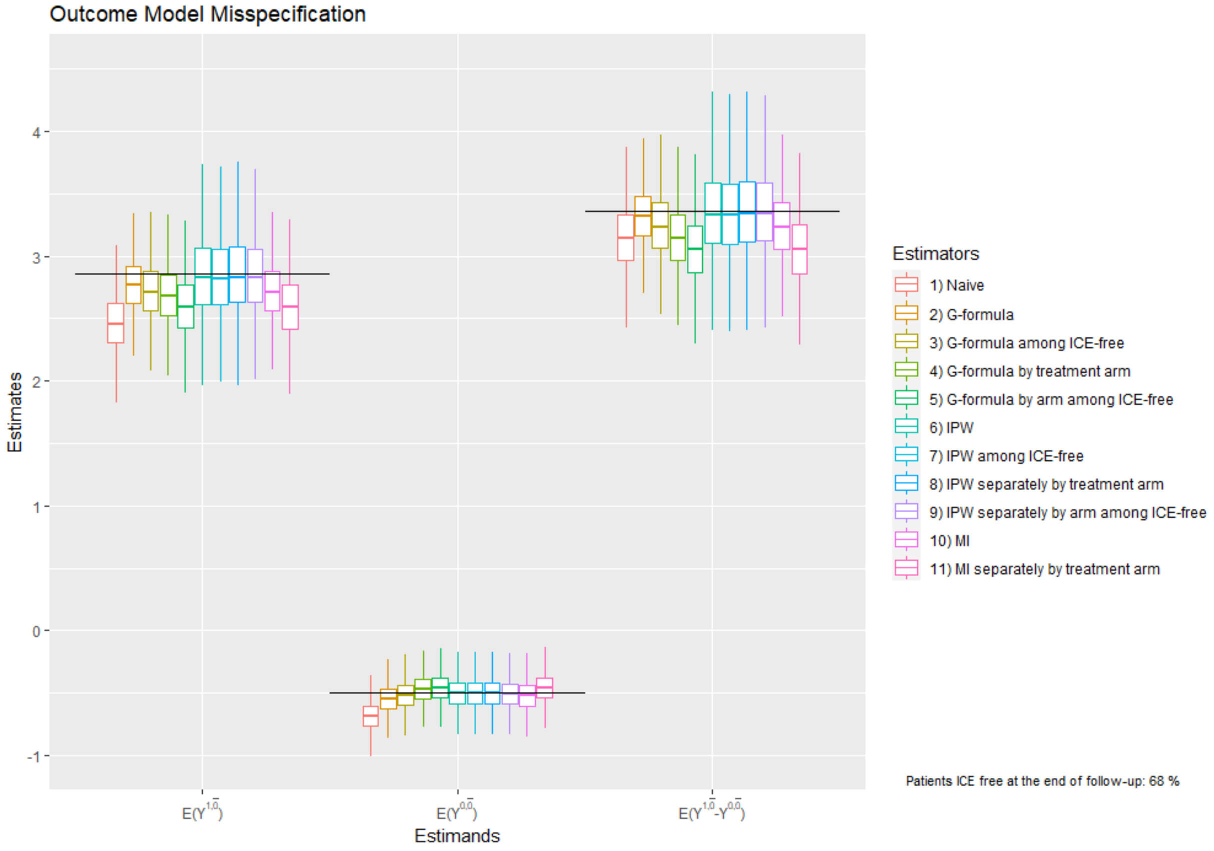

Figure 3: Simulation results showing the treatment effect as estimated by different methods, when the outcome model is misspecified.

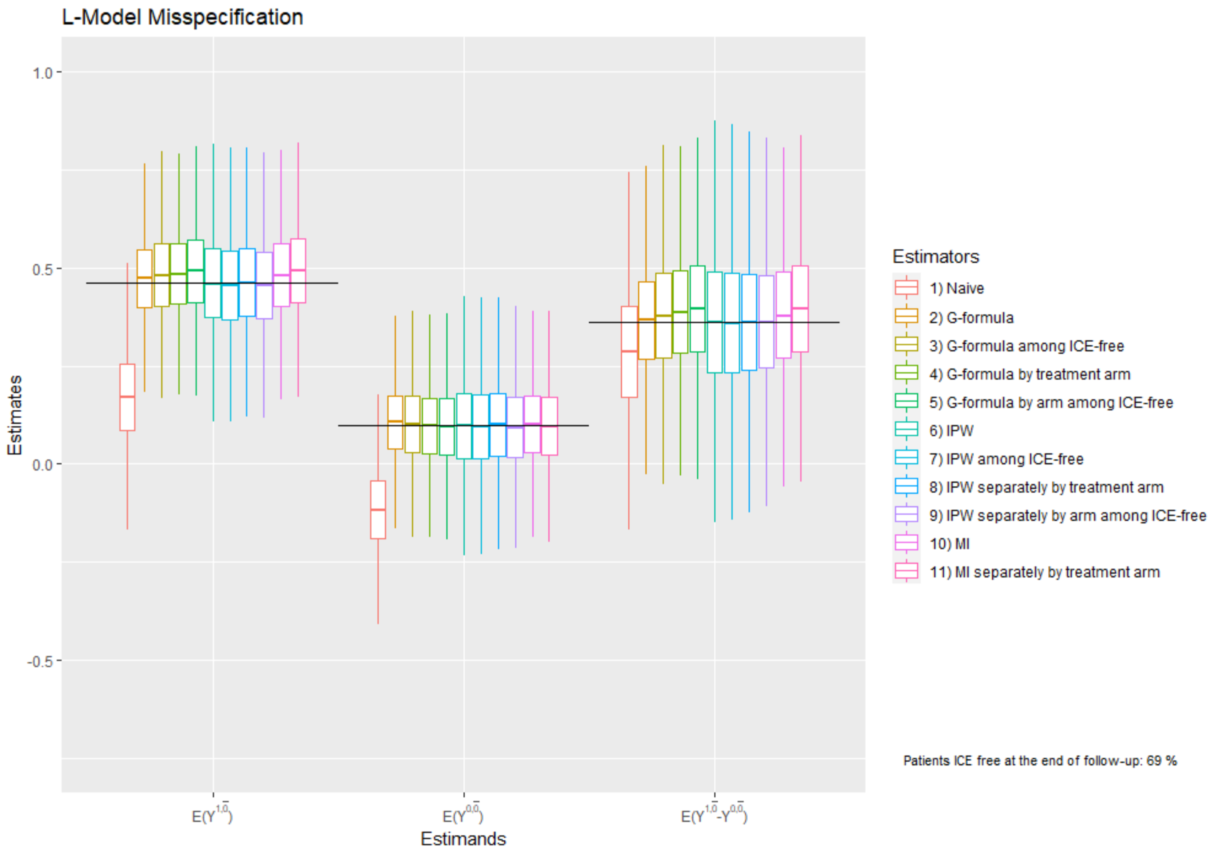

Figure 4: Simulation results showing the treatment effect as estimated by different methods, when the L-model is misspecified

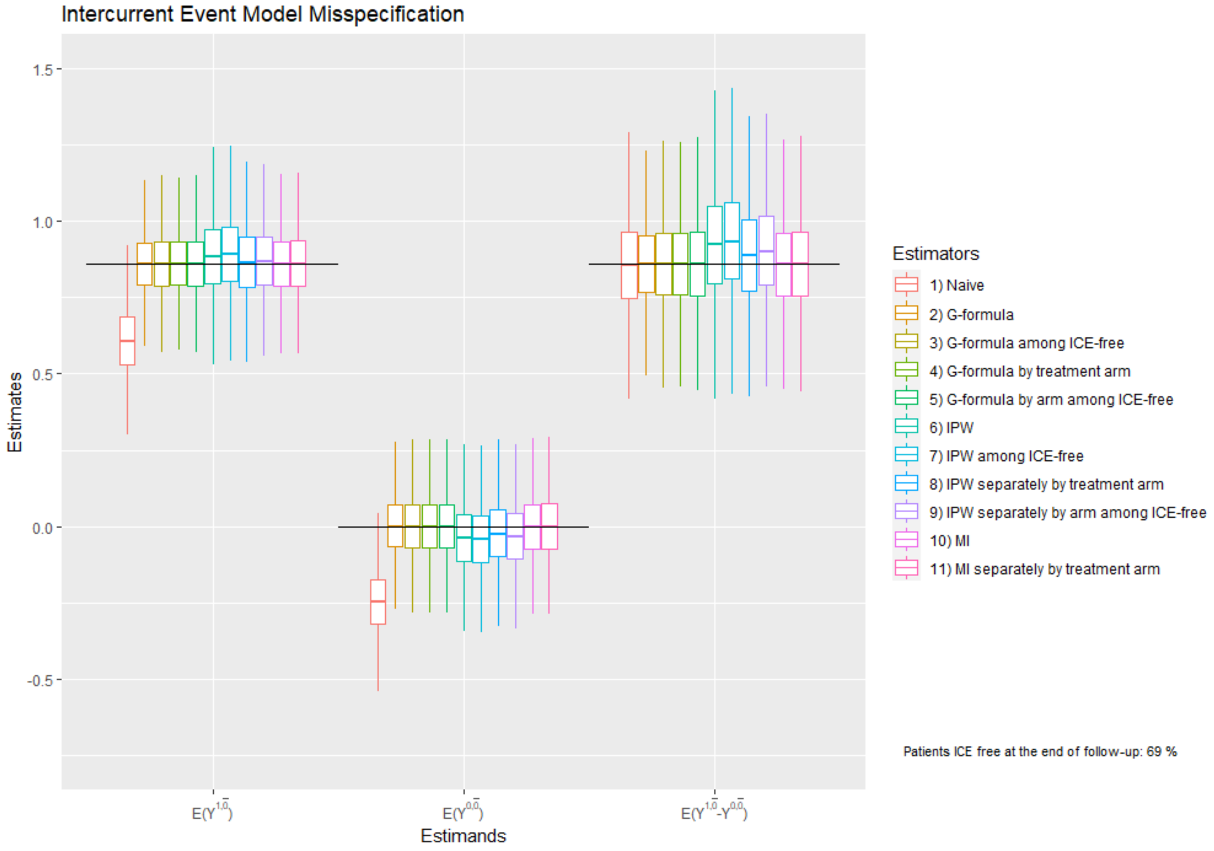

Figure 5: Simulation results showing the treatment effect as estimated by different methods, when the ICE model is misspecified

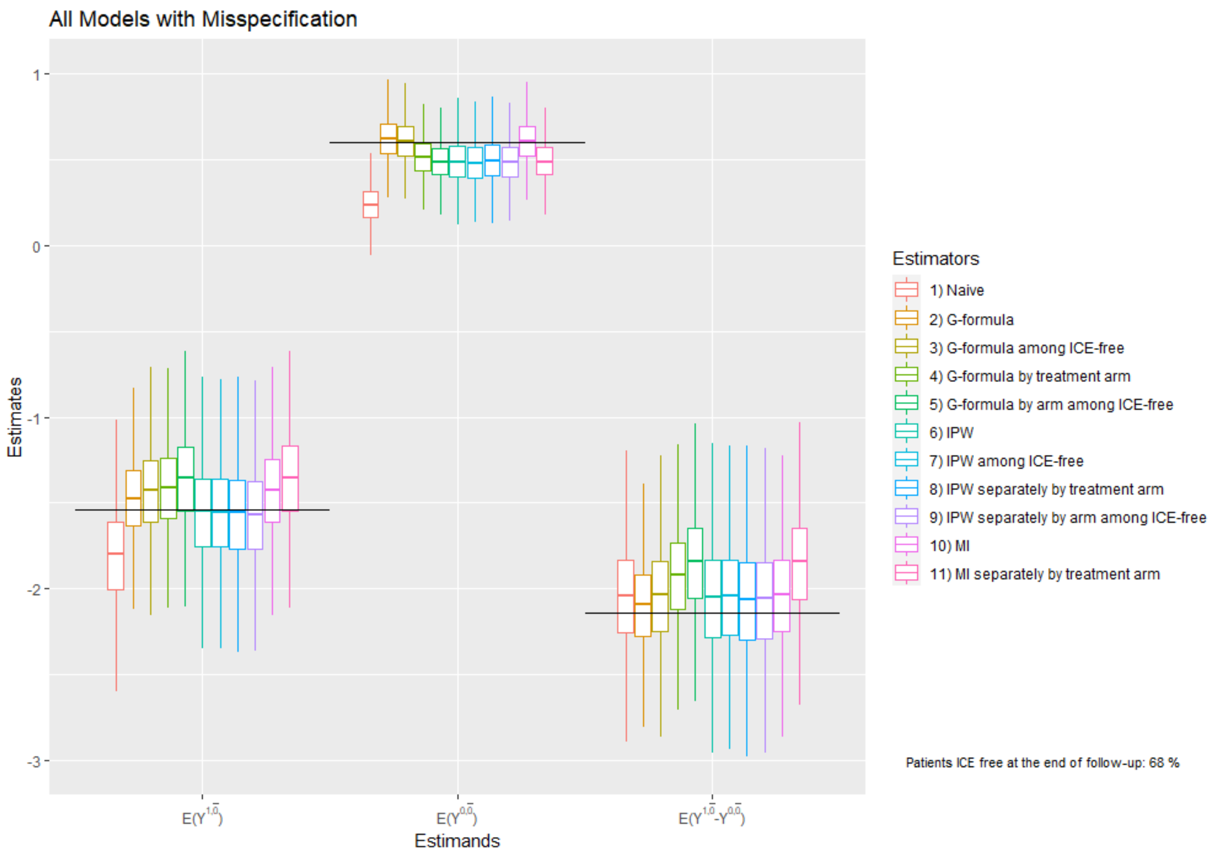

Figure 6: Simulation results showing the treatment effect as estimated by different methods, when the L, ICE and outcome models are all misspecified
